# A Generalized Approach for Evaluating the Mechanical Properties of Polymer Nanocomposites Reinforced with Spherical Fillers

**DOI:** 10.3390/nano11040830

**Published:** 2021-03-24

**Authors:** Julio Cesar Martinez-Garcia, Alexandre Serraïma-Ferrer, Aitor Lopeandía-Fernández, Marco Lattuada, Janak Sapkota, Javier Rodríguez-Viejo

**Affiliations:** 1 Department of Physics, Nanomaterials and Microsystems Group, GNaM, Universitat Autònoma de Barcelona, 08193 Bellaterra, Spain; aleexserra.as@gmail.com (A.S.-F.); aitor.lopeandia@uab.cat (A.L.-F.); 2 Catalan Institute of Nanoscience and Nanotechnology (ICN2), Campus Universitat Autonoma de Barcelona, 08193 Bellaterra, Spain; 3 Department of Chemistry, University of Fribourg, Chemin du Musée 9, Office 403, 1700 Fribourg, Switzerland; marco.lattuada@unifr.ch; 4 Institute of Polymer Processing, Montanuniversitaet Leoben, 8700 Leoben, Austria; 5 Research Centre of Applied Science and Technology, Tribhuvan University, Kirtipur 44600, Nepal

**Keywords:** percolation threshold, mechanical reinforcement, nanocomposites, spherical fillers, interphase, interphase modeling, polymer nanocomposites

## Abstract

In this work, the effective mechanical reinforcement of polymeric nanocomposites containing spherical particle fillers is predicted based on a generalized analytical three-phase-series-parallel model, considering the concepts of percolation and the interfacial glassy region. While the concept of percolation is solely taken as a contribution of the filler-network, we herein show that the glassy interphase between filler and matrix, which is often in the nanometers range, is also to be considered while interpreting enhanced mechanical properties of particulate filled polymeric nanocomposites. To demonstrate the relevance of the proposed generalized equation, we have fitted several experimental results which show a good agreement with theoretical predictions. Thus, the approach presented here can be valuable to elucidate new possible conceptual routes for the creation of new materials with fundamental technological applications and can open a new research avenue for future studies.

## 1. Introduction

The introduction of inorganic fillers into polymer matrixes has emerged as an attractive design approach for the creation of new materials with novel and often unique combinations of properties [1,2,3,4,5]. Fillers are important additives in polymeric materials that not only have the potential to alter several physical properties of polymer composites (e.g., mechanical, electrical, thermal, optical, photonic and magnetic), but also may also lead to cost reductions [6]. It has been shown that dramatic improvements in mechanical properties can be achieved by adding a small amount of nanofillers without sacrificing the low cost, ease of processing and the lightweight of the composite [7,8]. This has also served as a pioneering technological route for developing innovations with fundamental impact in broad industrial areas such as automobiles, household goods, vibration mounts etc. [9,10].

The material’s stiffness can be readily improved by adding either micro- or nanoparticles, since rigid inorganic particles generally have much higher stiffness than the polymer matrixes. The strength of composites strongly depends on the stress transfer between the particles and the matrix. For well bonded particles, the applied stress can be effectively transferred to the particles from the matrix, giving rise to a clear strength improvement known as mechanical reinforcement [11,12]. This is also manifested in nonlinear viscoelastic behaviors [13], commonly explained in terms of a breakdown of a ‘filler network’ under the influence of the filler particle volume fraction; the particle morphology, surface area and surface activity [14]. On the other hand, the works of Heinrich and Klüppel [15,16] also explain such a striking reinforcement effect based on the formation and breakdown of physical (van der Waals) bonds between the networking filler particles. However, this mechanism is known to be insufficient to account for the markedly enhanced tensile modulus in polymer nanocomposites. The precise physical mechanisms underpinning the observed reinforcement phenomena are still only partially understood, being a crucial and open scientific problem.

The calculation of the Young’s modulus in polymer composites created by adding spherical particles (e.g., micro-/nano-SiO_2_, Al_2_O_3_, CaCO_3_, carbon black and layered silicates), has been extensively analyzed in the literature based on two-phase models (fillers + polymer matrixes) [17]. Classical models as well as several empirical or semi-empirical equations have been developed to estimate their tensile modulus. For example, based on the consideration that the tensile modulus of the composites under low shear stress would behave similarly to the viscosity of a fluid, the classical Einstein’s equation [18] developed to describe the viscosity increase due to spherical particles in a dispersion was adapted in 1944 by Smallwood [19] to the field of filled elastomers assuming perfect adhesion between fillers and polymer matrixes. Further, the interactions between particle pairs were incorporated by Guth [20] providing the formula known as the Guth-Smallwood-Einstein equation written as:

(1) $$\;E_{c}=E_{m}\left(1+2.5\;\phi +14.1\phi^{2}\right)$$

where the composite and the polymer matrix modulus are defined by *E_c_* and *E_m_* respectively and *ϕ* is the particle volume fraction. 

The linear term in Equation (1) accounts for the stiffening effect of individual particles in terms of a constant of 2.5, associated with a geometric factor for spherical particles. The second power term is the contribution of particle interactions. The premise of the equivalence between shear stress and viscosity of a fluid has subsequently inspired the establishment of another two popular semi-empirical equations, the Kerner equation [21], which is expressed in terms of the Poisson ratio, approximately assumed to be (*υ* = 0.35) and written as:

(2) $$E_{c}=E_{m}\left(1+\frac{15\left(1-\upsilon \right)\;}{8-10\upsilon }\frac{\phi }{\left(1-\phi \right)}\right)$$

and the general Halpin and Tsai [22,23] equation, which for the case of spherical particles, is written as:

(3) $$E_{c}=E_{m}\left(\frac{1+\eta \phi \;}{1-\eta \phi }\right)$$

where = (*E_f_* − *E_m_*)/(*E_f_* + *E_m_*), *E_f_* and *E_m_* are the tensile moduli of the filler and polymer matrix, respectively.

Nielsen [24] modified the Kerner approach by postulating a general equation as a function of the particle packing fraction. Instead, the famous equation of Mooney [25] has introduced another modification to Einstein’s equation by introducing an s-parameter, defined as a crowding factor that reproduces Einstein’s equation at low volume fractions. Christensen and Lo [26] presented a simplified model for studying the mechanical reinforcement in polymer filled with hard spherical particles. Equations based on perfect adhesion between the phases have also been proposed by Counto [27] and Verbeek [28] and by Mori–Tanaka [29], whose micromechanical approach has already proved to be greatly successful in the prediction of the overall effective elastic moduli of composites. Other equations have been very well summarized in the review by Shao-Yun Fu et al. [30].

Despite the existence of these equations, their experimental validation shows that in general, they are not accurate enough. This issue is experimentally illustrated by several researchers [31] who faced challenges in finding an agreement between their experimental results and the theoretical predictions. The divergence of the fittings, either at a low filler volume fraction or at higher filler volume fractions, indicates that, in addition to the hydrodynamic reinforcement, the filler–filler interaction and polymer-filler interaction, there are other parameters to be considered. One of the fundamental aspects that is not considered by classical models to date is the formation of an interfacial glassy layer between the polymer matrix and nanofillers [32,33]. Although a practical and precise technique for the estimation of interfacial interactions or interphase characteristics has not been established to date, the existence of such an interphase has been experimentally and computationally revealed. For example: (i) based on NMR experiments on silica filled elastomer model systems, Berriot et al. [34,35,36] and others [37,38] observed a layer of immobilized segments at the particle surface (glassy layer), whose thickness varies with temperature. This result was in agreement with previous works by Struik [39,40], who found a glassy shell around particles in filled rubber; (ii) via torsional harmonic Atomic Force Microscopy (AFM) indentation, Meng Qu et al. [41] showed evidence of the existence of a particle interphase in hydrogenated nitrile butadiene rubber (HNBR)–carbon black composites, through direct visualization and measurement of their elastic properties; (iii) Lewis and Nielsen [42] indicated a surface layer containing an excess of matrix material, giving rise to a modulus increase as particle size decreases; (iv) Vollenberg and Heikens [43] observed an effective reinforcement for a polystyrene with fine silica and chalk particles by the formation of a more dense matrix in the interfacial region; (v) Takayanagi et al. [44] have noted the formation of microfibrils with diameters from 10 to 30 nm, which were more influential at the interphase boundaries than in the bulk system; (vi) based on large-scale dissipative particle dynamics simulations Gavrilov et al. [45] concluded that several sets of subchains in the polymer matrix around the filled particles have distinct properties and are deformed slightly more than in the unfilled matrix; (vii) using finite elements calculations, Gusev [46] indicated that the mechanical reinforcement can be explained micromechanically, by taking into account that the networking filler particles are joined by coating layers of immobilized rubbers. All these evidences clearly indicate that the strong interfacial interactions between polymer matrix and particles at the nanoscale will form an interphase, namely a third phase, which has different properties from both the matrix and the nanofiller phases and can depend on several other factors, such as the type of polymers, the presence of functional groups on the polymer and on the fillers and on the interactions between polymer-filler and filler-filler.

Analytical treatment of composites, including interphases, has received significant attention. For example, by solving for the stress field and effective bulk moduli of composites containing spherical particles, Lutz and Zimmerman [47] and Weng and Ding [48] explored the mechanical contributions of an interphase. Herve and Zaoui [49] proposed a model with an n-layered spherical inclusion embedded in an infinite matrix. Nie and Basaran [50] developed a series of parameterized equations from which bulk and shear elastic moduli could be calculated. However, the predictions of elastic properties were obtained by solving the elastic governing equations with high mathematical complexity. To minimize such complexity, Deng and Van Vliet [51] employed a micromechanical analytical approach termed interaction direct derivation (IDD) [52,53,54], to estimate the effective elastic properties of composites comprising spherical particles surrounded by mechanically distinct interphases, showing good correspondence with experimental results. In addition, it is important to remark on the prominent three-phase model developed by X. Ling Ji et al. [1]. Based on Takayanagi’s two-phase approach [44] and assuming a linear gradient distribution of the modulus of the interface, an analytical equation to calculate the Young’s modulus of polymer composites formed with spherical nanoparticles of radius,R, and thickness interphase,r, is derived. This yields a relationship of tensile moduli as a function of their nanoparticles content *E_c_ (ϕ)*, written as:

(4) $$E_{c}=E_{m}{\left[\left(1-\delta \right)+\frac{\delta -\gamma }{\left(1-\delta \right)+\frac{\delta \left(k-1\right)}{ln\left(k\right)}}+\frac{\gamma }{\left(1-\delta \right)+\frac{\left(\delta -\gamma \right)\left(k+1\right)}{2}+\gamma \frac{E_{f}}{E_{m}}}\right]}^{-1}$$

where $\delta =\sqrt{\left(1+\frac{r}{R}\right)\phi }$ and $\gamma =\sqrt{\phi }$.

The tensile moduli of the composite, polymer matrix, interphase and filler particles are denoted by *E_c_*, *E_m_*, *E_i_* and *E_f_* respectively where the k-parameter is *k* = *E_i_*/*E_m_*. This parameter takes values between the minimum case, *E_i_* = *E_m_*, i.e., *k* = 1, meaning no interphase contribution (*r* = 0, signifying that the volume fraction of the fillers is much greater than that of the interface region) where Equation (4) reduces to the classical Takayanagi two-phase model, and possible maximum values when *E_i_* = *E_f_*, meaning that 1 < *k* < *E_f_*/*E_m_*. 

Besides the above mentioned theoretical, empirical-semi empirical and micromechanical approaches, there are two important issues that have been overestimated: (i) to date, all developed approaches consider that 100% of the added particles will contribute into the mechanical reinforcement; however this will only occur if the particles aggregate/interact with each other. When this happens, a percolation network of particles will be formed at a critical volume fraction, the starting point of the contributions to the mechanical reinforcement. (ii) The interfacial glassy layer formation (also known as. the third phase) is directly correlated to the size and volume fraction of particles in addition to the nature of the polymer. These are two important factors that have been omitted in previous approaches. Hence, the purpose of the present paper is to develop a generalized and more complete theoretical approach to calculate the tensile modulus of polymer nanocomposites reinforced with spherical nanoparticles. 

Based on the three-phase series-parallel model of X. Ling Ji et al. [1] and the percolation approach of Schilling et al. [2], herein, we have developed a three-phase model, including both the percolation concepts and the glassy layer, as well as the colloidal glass transition. We firstly introduced the concept of the effective particles explaining the percolation concepts. We then present the generalized approach and briefly visualiz some representative cases. Finally, we experimentally validate the approach by using data of six polymer nanocomposites having unique properties and specific uses for technological applications.

## 2. Generalized Approach

To describe the nanoparticle–interphase–matrix composite, we assume that all particles will have radius R and an interphase with a uniform thickness r.

The volume fraction of the particles is defined as *ϕ* = *NV_p_*/*V*, where *N* is the number of particles, the single particle volume is *V_p_* and *V* is the total system volume. The consideration of the polydispersity effect is an issue, which can be added as a next step into our general approach, but in order to simplify we consider this aspect beyond the scope of this paper. The nanoparticle–interphase regions will be assumed as core–shell assemblies embedded in an infinite polymer matrix. All interfaces between particles and the surrounding matrix will be assumed to be perfectly bonded, thereby removing additional complexities. We will also consider the particles’ interactions as those of hard-spheres, meaning that they cannot interpenetrate.

### 2.1. Effective Particles Contributing to the Mechanical Reinforcement

By connecting polymer chains and filler particles, a network between fillers and polymers is created, which enhances the mechanical properties of nanocomposites. Above a certain volume fractions, the particles will form a percolating network giving rise to a stepwise change of their tensile modulus (mechanical reinforcement) observed upon crossing a critical point *ϕ_p_*, defined as the percolation volume fraction. This threshold will depend on several variables, such as the sizes, shapes and orientations of particles, with particle interface sizes being a fundamental parameter that cannot be overlooked. Its clarification will be fundamental for the precise understanding of the mechanical reinforcement. On the other hand, it should not be forgotten that as one increases the particle concentration, the system exhibits a dramatic increase in viscosity where upon crossing a critical volume fraction *ϕ_g_*, the particle movements are slow enough that it can be considered essentially frozen, leading to a glass transformation (colloidal glass transition), which was discussed already in 1980’s [55,56,57]. This effect must also be taken into account, especially for a precise characterization of the mechanical properties of the composite.

**Figure 1:**
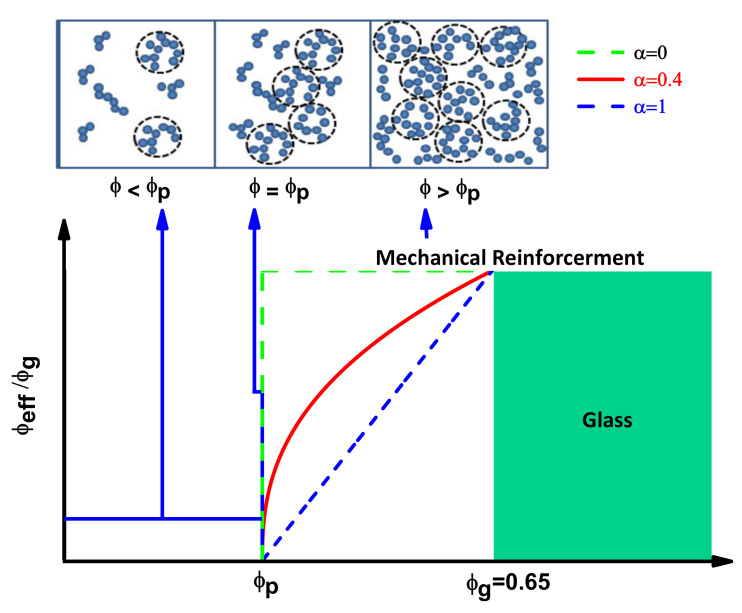
Effective particles contributing to the mechanical reinforcement.

For spherical particles, the glass transformation will be mainly reached at a specific concentration depending on the nature of the polymer and filler. For our model validation, we will set it as *ϕ_g_* ≈ 0.65, which is intrinsically correlated with the maximum density of occupancy of the spherical particles [58]. Above this volume fraction, fillers will not diffuse through the sample anymore and, hence, percolation will no longer be possible. This will imply that only a portion of particles *ϕ_eff_* = *Aϕ_g_* will effectively contribute to the formation of the percolating network within a restricted particle volume fraction domain (*ϕ_p_* < *ϕ* ≤ *ϕ_g_* *≈* 0.65), as is illustrated in Figure 1. 

The following three statements can be then formulated: (i) when the particle volume fraction (*ϕ*) is smaller than the percolation threshold *ϕ_p_*,the effective particles *ϕ_eff_* go to 0; (ii) when *ϕ* = *ϕ_g_*, *ϕ_eff_* will reach the maximum value at the glassy phase *ϕ_g_* and (iii) based on considerations pointed out by Ouali [59], the ratio *ϕ_eff_*/*ϕ_g_* can be described by a power law dependence, i.e., *A*(*ϕ* − *ϕ_p_*)*^α^* where *α* defines the percolation exponent. Based on that, the effective amount of particles contributing to the mechanical reinforcement can be found by the following equation (see Supplementary Materials):

(5) $$\;\phi_{eff}=\left\{\begin{array}{cc} 0 & :0\leq \phi \leq \phi_{p}\\ \phi_{g}{\left(\frac{\phi -\phi_{p}}{\phi_{g}-\phi_{p}}\right)}^{\alpha } & :\phi_{p}<\phi \leq \phi_{g} \end{array}\right.$$

As shown in Figure 1, three representative’ cases derived from Equation (5), green dotted line (*α* = 0), blue dotted line (*α* = 1), red full line (*α* = 0.4), are visualized, where the percolation volume fraction is denoted by *ϕ_p_*. If a glassy phase is immediately formed after the particles percolate, a step function will describe the process (*α* = 0), corresponding to a hypothetical extreme situation of composites formed with many particle interactions, where all the particles immediately become trapped.

The other limit case, *α* = 1, will take place due to weaker particle interactions, where the glassy phase will be reached in a uniform and slower linear manner. A more realistic situation will follow a pattern assertively modeled by a power law behavior with exponent 0 < *α* < 1, and the universal case *α* = 0.4 is exemplified. The percolation exponent will provide quantitative information about the dynamic aggregation of the particles as well as of the rapidity of the interphase glassy phase formation.

### 2.2. Percolation Threshold 

For calculating the percolation volume fraction, we have used here the recent prominent theoretical approach developed by Schilling et al. [2] where an analytical equation able to predict the percolation threshold from spheres to extremely slender particles was developed. For the case of composites formed with spherical particles of radius *R* and thickness *r*, the mentioned equation is reduced to the following relationships (see Supplementary Materials):

(6) $$\;\phi_{p}\left(r,R\right)=\frac{2\left(1+\xi_{sphere}\right)-2{\left(1+\frac{\xi_{sphere}}{2}\right)}^{\frac{1}{2}}}{3\left(1+\frac{2}{3}\xi_{sphere}\right)}$$

$$\xi_{sphere}=\frac{1}{4\left({\left(1+\left(r/R\right)\right)}^{3}-1\right)}$$

Two important trends can be elucidated: (1) composites formed of particles with thick interfaces in comparison with the particle radius (*r* ≫ *R*) will need fewer particles to initiate mechanical reinforcement, and therefore percolate at a lower particle volume fraction.

**Figure 2:**
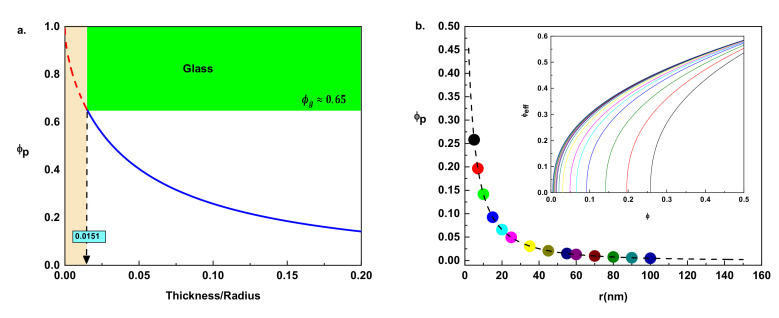
Percolation threshold: (**a**) numerical illustration of Equation (6) as the function of thickness and radius size effect. When the thickness of the particles is 1.5% of their radius, a hypothetical and extremely *R*-bound case will take place ϕp = ϕg. (**b**) A modeled situation for different composites formed with particles of *R* = 50 nm having different thicknesses, ranging from 5 nm (black point) to 100 nm (blue point) where the dotted line is the plot of Equation (6) in the entire *r*/*R* domain. The inset part of the right figure displays the corresponding values of the effective number of particles determined from Equation (5) where the power law exponent is assumed to be 0.4.

For example, in the hypothetical case of *r* = 2*R,* the percolation threshold will be 0.00478 (blue point of Figure 2a), meaning that the systems will percolate at only 1% of *ϕ_g_* to start the mechanical reinforcement, while composites with particles having smaller interphases in comparison with particle radius (*r* << *R*) will require a greater number of particles to interconnect with each other, leading to higher percolation volume fractions. The black point of Figure 2a illustrates the last situation, when particle thickness is 10% of particle radius, and the percolation threshold will be 0.26 where the systems will need a considerable amount of particles (40% of *ϕ_g_*) to start mechanical reinforcement. If the system percolates at the limiting case *ϕ_p_* = *ϕ_g_*, a hypothetical and totally unfeasible case will take places at a lower bound of r/R = 0.0151 (Figure 2b). The percolation threshold can never be greater than the glass particle volume fraction (0 ≤ *ϕ_p_* ≤ 0.65) meaning that the interphase thickness cannot be smaller than 1.5% of particle radius, for example in composites formed with particles on the micrometers scale (e.g., *R* = 1000 nm) the thickness will never be smaller than 15 nm. The mentioned situations are extreme cases, but how consistent such boundary predictions are in comparison with real situations must be understood.

Considering the AFM experimental results by Meng Qu et al. [41], a particle interphase thickness of *r* = 19 ± 8 nm was estimated in carbon black spherical particles having average radius particles of *R* = 56 ± 9 nm. Such a value will correspond to *r*/*R* = 0.33, which implies *ϕ_p_* = 0.08, perfectly consistent with the theoretical prediction. On the other hands, for spherocylindrical surfaces of carbon particle (diameters on the order of a nanometer), quantum mechanical treatment gives rise to a representative value of *r*/*R* = 0.2 [60], which means *ϕ_p_* = 0.14 which is also perfectly reliable, and this implies that Equation (6) can be a valuable and consistent approach to compute percolation thresholds in polymer composite systems formed with spherical particles and having glassy interphase regions.

### 2.3. Critical Percolation Exponent

According to the works of Stauffer and Aharony [61] and de Gennes [62], the percolation exponent is generally assumed as *α* = 0.4, even though there are some works that have considered the exponent as a free fitting parameter. For example, Bauhofer et al. [63] obtained *α* = 0.7 for polymeric nanocomposites of single walled carbon nanotubes, and Nawaz et al. [64] used *α* = 0.8 for graphene oxide elastomer composites. For interpreting the experimental tensile modulus of cellulose based composite data, it is also assumed that *α* = 0.4 [65,66], even though, in our opinion, it is difficult to accept such an exponent as universal. Considering that for each composite, we will only have a single exponent value, the following relationship can be directly derived from Equation (5):

(7) $$\alpha =\frac{dln\left[\phi_{eff}\right]}{dln\left[\phi -\phi_{p}\right]}$$

Considering the mathematic definition of the elasticity of a differentiable function [67], Equation (7) can be interpreted as the ratio of the percentage change in *ϕ_eff_* to the percentage change in *ϕ* − *ϕ_p_* of a composite or, equivalently, as the slope of an *ln*(*ϕ_eff_*) vs. *ln(ϕ* − *ϕ_p_)* plot (numerical example is plotted in Supporting Information). From the physical point of view, Equation (7) can also be understood as a Grüneisen parameter [68], which for molecular glasses is written in terms of their index of activation energy, which extremely valuable to elucidate the nonlinear thermal behavior of the glass transformation process [69,70,71]. Based on the aforementioned arguments, we can introduce a new interpretation of the percolation exponent as a measure of the aggregation dynamics of the particles, intrinsically related to the speed of the glassy phase formation. This will provide information about how fast or slow the vitreous phase can form, intrinsically correlated with the degree of strength of the interactions of the particles and their coupling/aggregations within the polymer matrix. If the exponent were universal, it would imply that regardless of the nature of the polymer matrix and the type of particles, the particles will always become trapped in the same manner, following a universal pattern curve (Equation (5) with a constant exponent). However, as pointed out in the Introduction, this process depends on several interconnected parameters, such as particle interface, type of particles, particle physical properties (charge values and their sign) and strength of the filler-filler and filler-matrix interactions, all of which will not necessarily take place in the same manner. For the aforementioned reasons, we will not consider the percolation exponent as universal, but as a fitting variable intrinsically coupled with the rest of the parameters.

### 2.4. Tensile Modulus 

The response to an applied stress in the composite will be schematically described by three phases connected to each other in series and in parallel, where the tensile modulus of the composite, polymer matrix interphase and filler particles will be denoted by *E_c_*, *E_m_*, *E_i_* and *E_f_*, respectively (Supporting Information Section S4). The modulus of the interfacial region will also be assumed, as in case of the X. Ling Ji et al. approach [1], by a linear gradient change in modulus between the polymer matrix and the surface of the particle, and is quantitatively described in terms of a k-parameter defined as (*k* = *E_i/_E_m_*). 

Based on the mentioned assumptions, for the case of a three-phase model (*r* > 0), we have conceptually incorporated Equations (5) and (6) into the main considerations of X. Ling Ji et al.’s approach [1], improving the calculation of the tensile modulus of the interface region (corner boundary effect) by assuming a linear gradient distribution of the modulus along the direction of the normalized vector *u =* (−√2/2,−√2/2). This gives rise to a correction to Equation (4), leading to the following general equation (see Supporting Information):

(8) $$E_{c}=E_{m}{\left[\left(1-\delta \right)+\frac{\delta -\gamma }{\left(1-\delta \right)+\left(\frac{k-1}{ln\left[k\right]}\right)\gamma +\left(\left(k+\frac{\sqrt{2}}{2}\left(k-1\right)\right)\left(\delta -\gamma \right)\right)}\;+\frac{\gamma }{\left(1-\delta \right)+\frac{\left(\delta -\gamma \right)\left(k+1\right)}{2}+\gamma \frac{E_{f}}{E_{m}}}\right]}^{-1}$$

$$\delta =\left\{\begin{array}{cc} 0 & :0\leq \phi \leq \phi_{p}\\ \sqrt{\left(1+\frac{r}{R}\right)\phi_{g}}{\left(\frac{\phi -\phi_{p}}{\phi_{g}-\phi_{p}}\right)}^{\alpha /2} & :\phi_{p}<\phi \leq \phi_{g} \end{array}\right.$$

$$\gamma =\left\{\begin{array}{cc} 0 & :0\leq \phi \leq \phi_{p}\\ {\left[\sqrt{\phi_{g}}\left(\frac{\phi -\phi_{p}}{\phi_{g}-\phi_{p}}\right)\right]}^{\alpha /2} & :\phi_{p}<\phi \leq \phi_{g} \end{array}\right.\;\;\;\;$$

where *ϕ_g_* ≈ 0.65, the radius of the particle will be (*R*) and the tensile modulus of the polymer matrix *E_m_* and the percolation threshold *ϕ_p_* will be determined from Equation (6). On the other hand, the thickness of the interphase (*r*), the rate of the interphase modulus *k*-parameter, the tensile modulus of the particles *E_f_* and the percolation exponent α will be considered as fitting model parameters.

**Figure 3:**
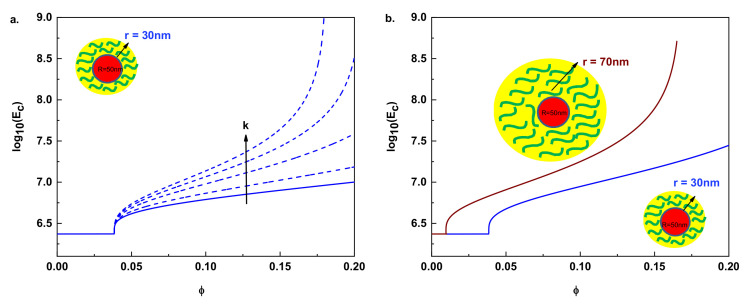
Numerical evaluation of the (**a**) hardness and (**b**) size effects of the interphase in the mechanical reinforcement for hypothetical composites. The lines are the plot of Equation (8) with Equation (5). The percolation exponent for both figures is assumed as 0.4, *k* parameters in (**b**) as 1.5, the tensile modulus of the filler and the matrix as *E_f_* = 1 × 10^11^ Pa and *E_m_* = 2.4 MPa respectively.

As we see from Equation (8), the mechanical reinforcement (*E_c_* > *E_m_*) will take place only above the percolation threshold (*ϕ_p_* < *ϕ* ≤ *ϕ_g_*), manifested by a step-wise change behavior of *E_c_*. Below the mentioned threshold (0 ≤ *ϕ* < *ϕ_p_*), the tensile modulus of the composites will be the same as that of the polymer matrix *E_c_* = *E_m_* (*δ* = *γ* = 0). This trend is numerically visualized in Figure 3 by modelling the hardness (left) and size (right) effects of the particle interphase in the mechanical reinforcement for hypothetical composites. The lines correspond to the plot of Equation (8) with Equation (6). The left figure visualizes the cases of different composites formed with particles of radius *R* = 50 nm and thickness *r* = 30 nm. When k changes from 1 to 5, the tensile modulus of the composite *E_c_* will gradually increase, and, especially for *k* > 3, the slope of the curve (*d*log*E_c_/d_ϕ_*) will change drastically giving rise to a higher mechanical reinforcement. The right part of Figure 3 shows the case of two different composites formed by the addition of particles with the same radius and different thickness interphases into the same polymer matrix. From this modeled situation, we can clearly see that composites formed with particles with large interfacial thicknesses (brown line *r* = 70 nm) will need fewer particles in comparison to the particles with smaller thicknesses to initiate mechanical reinforcement and, therefore, will percolate at a lower particle volume fraction. This will give rise to an increase in modulus for the resulting composite, as compared to the smaller thickness case (blue line), which shows that when the particle size is in the nanoscale range, the interfacial region greatly affects *E_c_*. Undoubtedly, the four fundamental discussed effects (particles and interphase size, tensile modulus, ratio of the interface, and percolation exponent) will have a dominant influence on *E_c_* and validation of these parameters with experimental data is the ultimate goal.

## 3. Model Validation and Discussion

**Figure 4:**
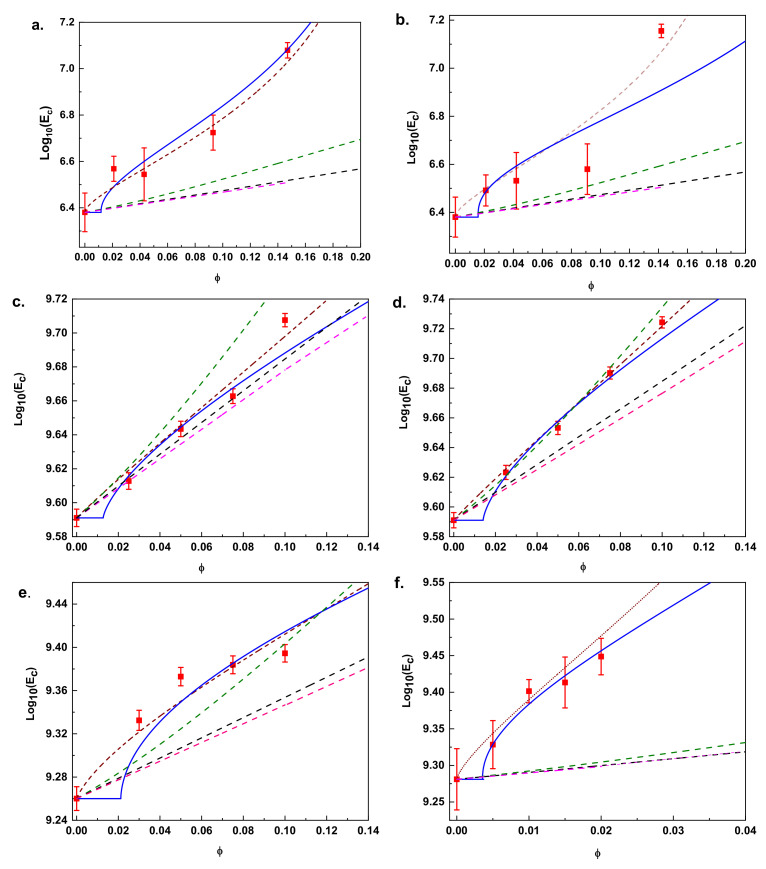
Comparison between experimentally obtained (red squares) Young’s modulus vs theoretical prediction using different approaches: our generalized approach (Equation (8), solid blue line), X. Ling Ji model (Equation (4), brown dashed line), Guth-Smallwood-Einstein equation (Equation (1), pink dashed line), Kerner equation (Equation (2), green dashed line) and Halpin and Tsai equation (Equation (3), black dashed line) for (**a**) polyolefin/carbon black (**b**) polyolefin/fumed silica (**c**) PEEK/Al_2_O_3_ (**d**) PEEK/SiO_2_ (**e**) PTMHMTA/TiO_2_ and (**f**) P(MMA-MTC)/SiO_2_.

In order to test the consistency of the developed approach, we have collected experimental data describing the variations of the tensile modulus as a function of their nanoparticle content *E_c_* (*ϕ*). The data correspond to six nanocomposites having unique properties and specific performances for technological applications (see details in data information). They are extracted from dynamic mechanical thermal analysis (DMTA) experiment at temperatures above the glass transition temperature *T_g_*, where a higher modulus is experimentally observed when increasing the filler content. A common trend presented in these types of polymer composites is observed. At lower filler content, the modulus of the composites is only slightly higher than that of the unfilled material. However, a higher modulus is experimentally reached with increasing filler content. In order to explain this, we have fitted the experimental data with five model equations which are plotted in Figure 4. The fitting curves in Figure 4 correspond to: (1) our general approach (Equation (8), solid blue line), (2) the X. Ling Ji et al. model (Equation (4), brown dashed line), (3) the Guth-Smallwood-Einstein equation (Equation (1), pink dashed line), (4) the Kerner equation (Equation (2), green dashed line) and (5) the Halpin and Tsai equation (Equation (3), black dashed line). Table 1 summarizes the parameters (e.g., interphase size, *r*, modulus ratio of the interface, *k*, percolation exponent, *α*, and modulus of the fillers, *E_f_*) corresponding to the fitting of Equation (8) and Equation (4) respectively. 

It is clearly shown that at low filler content, the fittings curves of Equations (1)–(3) are relatively close to the experimental results, especially for the data shown in Figure 4c,d, however, as the filler content increases, the experimental tensile modulus becomes much higher than the predicted value. A pronounced nonlinear behavior of *E_c_*(*ϕ*) is reached at a higher volume fraction of fillers, indicating a clear inconsistency of the equations. This implies that, in addition to hydrodynamic reinforcement, both filler−filler interaction and polymer−filler interaction, and the particle interfacial effect, will contribute to the improvement of mechanical properties of the composites and should be considered.

The fitting of Equation (4) yields a good match in comparison with the classical equations, providing in principle a physical explanation for the higher storage modulus reached with increasing filler content. If we consider the acceptable mathematical fitting correspondence of Equation (4) in comparison with the fitting of Equations (1)–(3), we could think that the mentioned inconsistency is clarified. However, we should keep in mind that, besides Equation (4) and in all of the theoretical, empirical/semi-empirical and micromechanical approaches developed to date, two important effects have never been included, (i) the formation of percolation networks (ii) the colloidal glass transition. In addition, Equation (4) does not account for the corner boundary effect (see Supplementary Materials) into the tensile modulus of the interface region. Here, we have incorporated these important effects into our generalized approach Equation (8).

The corresponding fitting curves to our general model Equation (8) are illustrated in Figure 4. These show a remarkably good fitting quality in comparison with the classical Equations (1)–(3). On the other hand, at a higher particle content, a good correspondence between our model and the three-phase X. Ling Ji et al. approach [1] is reached, however, a remarkable difference is noted at a lower filler content. Stepwise reinforcement behavior of *E_c_* is manifested above the percolation threshold (*ϕ_p_* < *ϕ* ≤ *ϕ_g_*), which is true for all six composites.

Below the mentioned threshold, the tensile modulus of the composites will increase minimally. The percolation threshold will strongly depend on the particle size, *R*, and interphase thickness, *r*, as we have previously discussed in detail in a previous section. As we can see in Table 1, the determined fitting *r*-values from our model are similar to those determined by Equation (4) and are in the order of the measured interphase thickness [41]. 

As we discussed before, the percolation threshold will follow a *ϕ* ≈ (*r*/*R*)^−1^ tendency, meaning that composites formed of particles with large thickness interfaces in comparison with particle radius will need fewer particles to initiate mechanical reinforcement and therefore percolate at a lower volume particle fraction. Fitting the data of Figure 4f corresponds to a special case where composites were intentionally formed with a special polymer matrix where, with only 1 wt.% of particle content, a considerable enhancement of the mechanical reinforcement was achieved, while other composites need 10% particle content to considerably increase their mechanical properties. On the other hand, we can also see from Table 1 that the addition of particles with different physical properties in the same polymeric matrix will yield different percolation threshold values. Data in Figure 4a,b lead to *ϕ_p_* = 0.0117 and 0.0153 for carbon black and fumed silicate, respectively, with a discrepancy of 31%, while data in Figure 4c,d lead to *ϕ_p_* = 0.0127 and 0.0141 for Al_2_O_3_ and SiO_2_, respectively, having a discrepancy of 11%. Since the siloxane and silanol groups on the surface of the silica particles are hydrophilic in nature, attractive filler−filler interactions are strong due to the hydrogen bonds between silica particles. Thus, silica particles often form larger agglomerates that will lead to inhomogeneous filler distributions, making the dispersion of silica particles more difficult than the dispersion of other particles, such as carbon black and Al_2_O_3_. This implies that composites formed with silicate particles will have a tendency to reach higher percolation threshold values. The lower compatibility of spherical Al_2_O_3_ and SiO_2_ particles in PEEK could be the reason for the lower discrepancy of the percolation threshold. On the other hand, the dispersion of silica particles in polyolefins offers more resistance than the case of carbon black particles, leading to a higher percolation threshold difference. These data show that the formation of some percolating filler structures will affect the modulus of the composites and their effect should not be omitted. 

The modulus ratio of the interface, *k*-parameter will also be intrinsically correlated with the percolation effect. As we can see in Table 1, the values of the *k*-parameter obtained from the fitting of Equation (4) are higher than those obtained by our generalized equation. We can explain this because, although the determined fitting *r*-values from Equation (8) are similar to those determined from Equation (4), the X. Ling Ji et al. model [1] considers that 100% of the particles contribute to the mechanical reinforcement while, conversely, our model considers that only an effective amount of *ϕ_g_* will contribute. This will imply that the area of the total surface formed as a result of adding the individual interface thicknesses of each particle in the effective group of particles will be smaller than the area of the group formed by the total amount of particles. An ineffective extra interface thickness is hidden behind the *k*-values obtained from Equation (4), which gives rise to higher values of the *k*-parameter. 

Related to the assumption of a universal power law exponent α, we can see in Table 1 that the power law exponents give rise to different values, ranging from 0.63–0.72, which are considerably higher than the value of 0.4 predicted by Staugger and De Gennes [61,62]. As we have pointed out before, the percolation exponent provides information about how fast or slow the vitreous phase can form, which is intrinsically correlated with the degree of strength of the interactions of the particles and their coupling/aggregations within the polymer matrix. Weaker particle interconnections, such as those of nanospheres, will lead to higher values of the power law exponent in comparison with nanorods [11]. 

On the other hand, the lower compatibility of spherical Al_2_O_3_ and SiO_2_ particles in PEEK could be the reason why both composites have the same power law exponent of 0.74, irrespective of other differences [12]. Conversely, for the case of data in Figure 4a,b, the power law exponents are different, with the smallest values being the case of fume silicate. This could be justified due to the strong hydrogen bond interactions between silica particles, which lead to a faster interphase formation, resulting in a lower percolation exponent. In regard to the values of the tensile modulus of the fillers *E_f_*, we can also see in Table 1 that for each model equation, the obtained values are different, and the reason is because X. Ling Ji et al.’s approach (Equation (4)) considers an ineffective excess of material which does not contribute to the mechanical reinforcement below the percolation threshold (0 ≤ *ϕ* < *ϕ_p_*) and above the maximum density of occupancy of the spherical particles (*ϕ_g_* < *ϕ* ≤ 1). The obtained values are also considerably higher than those of the matrix *E_m_*, although variations of *E_f_*/*E_m_* will have only slight effects on the modulus of the composite *E_c_*.

## 4. Conclusions

In summary, we have demonstrated that the mechanical reinforcement of polymeric nanocomposites containing spherical nanoparticle fillers is dependent on the percolation threshold of filler, i.e., filler-filler network and glassy interphase between the polymer and the filler, meaning polymer-matrix interphase. The generalized equation proposed here represents the most complete three-phase model developed to date to account for the Young’s modulus in polymers composites formed with spherical nanoparticles. For the first time, both the percolation effect and the geometrical constraint of the maximum occupancy of spherical particles are incorporated into a three-phase approach to accurately evaluate the mechanical reinforcement of these composites. Our model was validated with experimental data of six polymer nanocomposites having unique properties and specific industrial performances, showing good agreement with the theoretical considerations. Thus, the approach presented here can be valuable to elucidate new possible conceptual routes for the creation of materials with unique technological applications, and can open a new a research avenue for future studies.

## Figures and Tables

**Table 1:** The samples, their characteristics and the calculated interphase properties.

			Our Model						X. Ling Ji et al. Model [1]		
No.	Composite [ref.]	*R* (nm)	*R* (nm)	*E_m_* (GPa)	*K = E_i_/E_m_*	*E_f_* (GPa)	α	$\phi_{p}$	*R* (nm)	*K = E_i_/E_m_*	*E_f_* (GPa)
1	Polyolefin ^1^/CB [31]	50	51	2.4 × 10^−3^	1.66	364	0.75	0.0117	59	2.76	579
2	Polyolefin ^1^/fumed silica [31]	7.5	8	2.4 × 10^−3^	1.43	4.3	0.63	0.0153	8	4.09	4.1
3	PEEK ^2^/Al_2_O_3_ [72]	15	15	3.9	4.37	19.2	0.74	0.0127	14	7.22	15.8
4	PEEK ^2^/SiO_2_ [72]	15	17	3.9	4.76	16	0.72	0.0141	15	7.72	17
5	PTMHMTA ^3^/TiO_2_ [73]	4.5	4	1.82	2.04	9	0.64	0.0212	4	2.22	24.9
6	P(MMA-MTC) ^4^/SiO_2_ [74]	10	23	1.91	2.35	428	0.72	0.0036	21	5.59	271

^1^: Carboxy-telechelic polyolefin prepolymers. ^2^: poly(ether ether ketone). ^3^: poly(trimethyl hexamethylene terephthalamide). ^4^: methyl methacrylate copolymerized with 2-(methacryloyloxy)ethyl trimethyl ammonium chloride comonomer.

## Data Availability

The data presented in this study are available on request from the corresponding author. The data are not publicly available as the data also forms part of ongoing study.

## Supplementary Materials

The following are available online at https://www.mdpi.com/2079-4991/11/4/830/s1, S1: Experimental data, S2: Derivation of Equation (5), S3: Derivation of Equation (6), S4: Derivation of Equation (7) and numerical example, S5: Derivation of Equation (8).
