# Supplementary material for: A Generalized Approach for Evaluating the Mechanical Properties of Polymer Nanocomposites Reinforced with Spherical Fillers
Source: Nanomaterials (Basel). 2021 Mar 24;11(4):830. doi: 10.3390/nano11040830 (PMC8063949; doi:10.3390/nano11040830)
Supplement: Supplementary file 1 [file nanomaterials-11-00830-s001.pdf]

nanomaterials

Supporting Information

A Generalized Approach for Evaluating the Mechanical Properties of Polymer Nanocomposites Reinforced with Spherical Fillers

J. C. Martinez-Garcia[1,*], A. Serraïma-Ferrer[1], A. Lopeandía-Fernández[1,2], M. Lattuada[3], J. Sapkota[4,5,*], and J. Rodríguez-Viejo[1,2*]

[1] Department of Physics, Nanomaterials and Microsystems Group, GNaM, Universitat Autònoma de Barcelona, 08193 Bellaterra, Spain; juliocesar.martinez@uab.cat (J.C.M.G.); aleexserra.as@gmail.com (A.S.F.); aitor.lopeandia@uab.cat(A.L.F.); javier.rodriguez@uab.cat (J.R.V.)
[2] Catalan Institute of Nanoscience and Nanotechnology (ICN2), Campus Universitat Autonoma de Barcelona, Bellaterra 08193, Spain; aitor.lopeandia@uab.cat(A.L.F.); javier.rodriguez@uab.cat (J.R.V.)
[3] Department of Chemistry, University of Fribourg, Chemin du Musée 9, Office 403 1700 Fribourg, Switzerland; marco.lattuada@unifr.ch (M.L.)
[4] Research Centre of Applied Science and Technology, Tribhuvan University, Kirtipur 44600, Nepal
[5] Institute of Polymer Processing, Montanuniversitaet Leoben, Leoben, 8700, Austria; sapkota.janak@outlook.com (J.S.)
* Correspondence: juliocesar.martinez@uab.cat (JCMG); sapkota.janak@outlook.com (J.S.); javier.rodriguez@uab.cat (J.R.V.)

S1. Experimental data

Data of Fig 4(a,b) in manuscript are taken from taken from Ref. [1], for the Polyolefin/Carbon black and Polyolefin/fumed silica nanocomposites. Composites were formed with the polymer matrix. Carboxy-telechelic polyolefin prepolymers were synthesized in the presence of fumed silica and carbon black fillers to yield polyolefin elastomers with significantly enhanced mechanical properties. The filled elastomers based on polyolefin prepolymer have excellent hydrophobicity, a wide operating temperature range, and a high elongation at break and are used for a wide range of applications, including sealing and joint

technologies. The resulting nanocomposites with only 4 wt.% of inclusions of carbon black or fumed silica nanoparticles exhibit 40–50% Young's modulus improvement. [1]

Data of Fig 4 (c,b) in manuscript are taken from Ref. [2], corresponds to poly(ether-ether-ketone) (PEEK) composites reinforced by nanosized SiO_2 and $Al2O_3$ fillers (PEEK/Al_2O_3 and PEEK/SiO_2). The inclusion of much cheaper (in comparison with carbon nanotubes CNT) nano SiO_2 or Al_2O_3 particles (with diameters ~15–30 nm) into PEEK is of basic interest for the purposes of processability and mechanical enhancement. The resulting nanocomposites with 10 wt.% SiO_2 or Al_2O_3 nanoparticles exhibit Young's modulus improvement of 30%. [2]

Data of fig 4(e) corresponds to polyamide–titania nanocomposites (PTMHMTA/TiO_2), taken from Ref. [3]. Polyamides are the first engineering thermoplastic polymers ever commercially produced. These polymers have a lot of applications as fibers, amorphous and crystalline plastics, and adhesives. On the other hand, titania has high melting point, resistance to attack by acids and alkalis and good mechanical properties to reinforce the polyamide matrix. At it is clearly showed when the TiO_2 content is 10 wt%, the Young's modulus of PTMHMTA/TiO_2 increases by 36% compared to corresponding of PTMHMTA. [3]

Data of fig 4(f) corresponds to the composite (P(MMA-co-MTC)/SiO_2) formed with SiO_2 nanoparticles without surface modification, taken from Ref. [4]. The polymer matrix was intentionally assembled by a promising method to improve the particle dispersion as well as their interfacial adhesion through electrostatic interaction without surface modification. The inclusion of 10 wt% of cationic functional comonomer 2-(methacryloyloxy) ethyltrimethylammonium chloride (MTC) into polymer matrix methyl methacrylate (PMMA) optimize considerable their mechanical reinforcement. As is clearly showed at the figure 4(f) when the MTC content is 10 wt% and SiO_2 content is only 1 wt%, the Young's modulus of P(MMA-co-MTC)/SiO_2 increases by 35% compared to corresponding P(MMA-co-MTC) which represent an optimal experimental route to produce materials having less costly with higher application. [4]

S.2 Derivation of eq.5

On the basis of percolation concepts as discussed in main text, the ratio $\phi_{\mathrm{eff}}/\phi_{\mathrm{g}}$ can be estimated by the following relationship:

$$\phi_{\mathrm{eff}}/\phi_{\mathrm{g}} = A(\phi - \phi_{\mathrm{p}})^{\alpha} \tag{s2.1}$$

when $\phi \to \phi_g$ the effective fraction of particles $\phi_{\mathrm{eff}} \to \phi_g$ leads to:

$$A = 1/(\phi_{\mathrm{g}} - \phi_{\mathrm{p}})^{\alpha} \tag{s2.2}$$

Substituting s2.2 into s2.1:

$$\phi_{\mathrm{eff}} = \phi_g \left(\frac{\phi-\phi_p}{\phi_g-\phi_p}\right)^{\alpha} \qquad \text{(s2.3)}$$

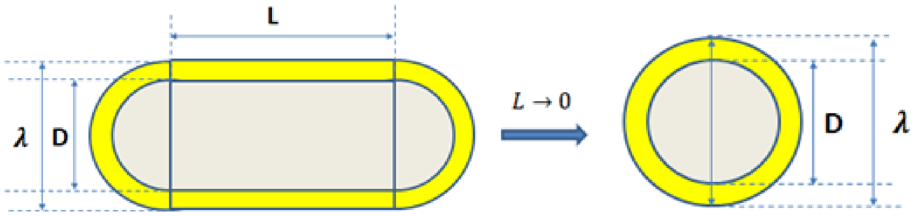

Figure S2. Schematic illustration of the spherical transformation. The diameter of the hard core, the length of the cylinder and the contact shell are denoted by D, L and λ respectively. The spherical case is recovered when $L \to 0$ which gives rises $\alpha = (r/R)$ being r the particle thickness interface and R the radius of the particle.

S.3 Derivation of eq.6b

The theoretical approach developed by Schilling *et al* [5] gives rise to the following general equation for the percolation threshold:

$$\phi_p(\gamma,\alpha) = \frac{2\left(1+\xi(\gamma,\alpha)\right)-2\left(1+\frac{\xi(\gamma,\alpha)}{2}\right)^{\frac{1}{2}}}{3\left(1+\frac{2}{3}\xi(\gamma,\alpha)\right)} \qquad \text{(s3.1)}$$

$$\xi(\gamma,\alpha) = \frac{\left(1+\frac{2}{3}\left(\frac{1}{\gamma}\right)\right)\frac{1}{\gamma}}{\frac{8}{3\gamma^2}\left((1+\alpha)^3-1\right)+\frac{4}{\gamma}\left((1+\alpha)^3-1\right)+\alpha} \qquad \text{(s3.2)}$$

where $\gamma = \frac{L}{D}$ defines the aspect ratio of the spherocylindrical particles and the connectivity variable is defined as $\alpha = \frac{\lambda}{D} - 1$ (see figure S3).

Eq.6b of the manuscript is obtained by passing the limit $L \to 0$ in eq (s3.2) which results in:

$$\xi_{sphere} = \lim_{\gamma \to 0} \xi(\gamma, \alpha) = \frac{2}{8((1+\alpha)^3-1)} = \frac{1}{4\left(\left(1+(r/R)\right)^3-1\right)} \quad \text{(s3.3)}$$

S.4 Derivation of eq.7 and numerical example

Applying Napierian logarithm in both sides of eqs2.3 yields:

$$ln(\phi_{\mathrm{eff}}) = ln(\phi_{\mathrm{g}}) + \alpha ln(\phi - \phi_{\mathrm{p}}) - \alpha ln(\phi_{\mathrm{g}} - \phi_{\mathrm{p}}) \quad \text{(s4.1)}$$

Differentiating with respect to ϕ

$$\frac{dln(\phi_{\mathrm{eff}})}{d\phi} = \frac{\alpha}{\phi-\phi_{\mathrm{p}}} + \frac{\mathrm{d}\alpha}{d\phi} ln(\phi - \phi_{\mathrm{p}}) \quad \text{(s4.2)}$$

For each composite we will only have a single exponent value, i.e. the percolation exponent will not depend of the particle concentration $(d\alpha/d\phi) \to 0$ and consequently:

$$\alpha = \frac{dln(\phi_{\mathrm{eff}})}{\frac{d\phi}{\phi-\phi_{\mathrm{p}}}} = \frac{dln(\phi_{\mathrm{eff}})}{dln(\phi-\phi_{\mathrm{p}})} \quad \text{(s4.3)}$$

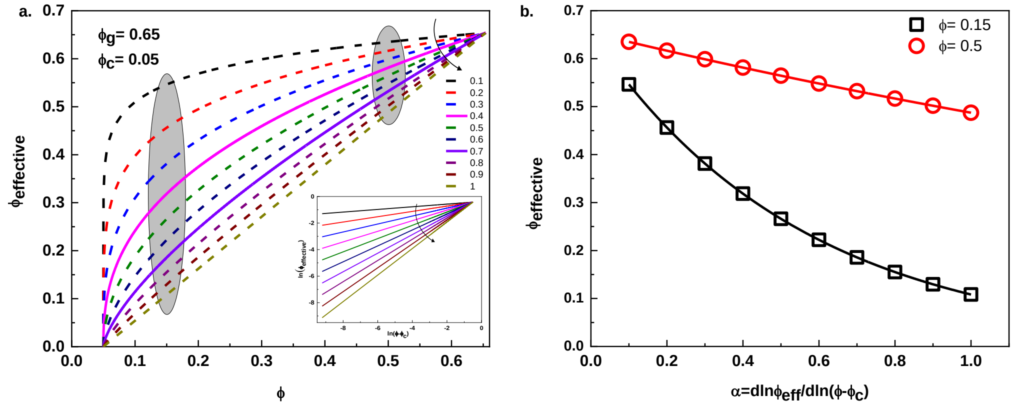

Figure S3. Numerical evaluations of eq.6 and eq.7 of the manuscript. The left part of the figure shows the evaluation of eq.6 for a particular case ϕ_p=0.05 and different values of α-exponent ranging from 0.1.to 1 and the right part illustrates the influence of the α-exponent into the effective numbers of particles for two specific situations: (1) close ($\phi = 0.15$) and far ($\phi = 0.5$) to the percolation threshold point. The insect part $ln\phi_{eff}$ vs $ln(\phi - \phi_p)$ visualizes the interpretation α-exponent is terms of the slope changes intrinsically related with the speediness of the glass transformation.

S.5 Derivation of eq.8

The eq.8 of the manuscript has been derived mainly based on the X. Ling Ji *et al* approach [6] however, here, two important improvements have been introduced: (1) Correction to the calculation of the Young modulus of the particles interphase and (2) Introduction of the percolation effect.

S.5.1) Correction of eq.4

As is shown in Fig.S4, we have evaluated the composite response to an applied stress ($T = F_T/A_{ST}$) as three phases connected in series A, B, and C, where the

regions B and C are analysed as sub-regions connected in parallel: (1) region B by two sections (polymer matrix (m) + interphase (i_B)) and (2) region C by three sections (polymer matrix (m) + interphase (i_C)+ effective dispersed phase). The red colour part in Fig S4 defines the effective volume fraction contributing into the mechanical reinforcement and F_T and A_{sT} the total applied force and total cross sectional area respectively.

By applying a gradual load to the composite, the total Yong modulus can be calculated as the slope of the linear stress–strain curve refers to the relationship between their axial normal stress and axial normal strain respectively which gives rises:

$$E_T = \frac{(F_T/A_{sT})}{(\Delta l_T/l_{0T})} = \frac{F_T l_{0T}}{\Delta l_T A_{sT}} \qquad \text{(s5.1)}$$

being Δl_T the total deformation of the composite and l_{0T} denotes the original dimensions of the sample before loaded. Consequently the Young modulus of the regions A,B and C will be written as (see Figs5A):

$$E_A = \frac{F_A l_{0A}}{\Delta l_A A_{sA}} \qquad E_B = \frac{F_B l_{0B}}{\Delta l_B A_{sB}} \qquad E_C = \frac{F_C l_{0C}}{\Delta l_C A_{sC}} \qquad \text{(s5.2)}$$

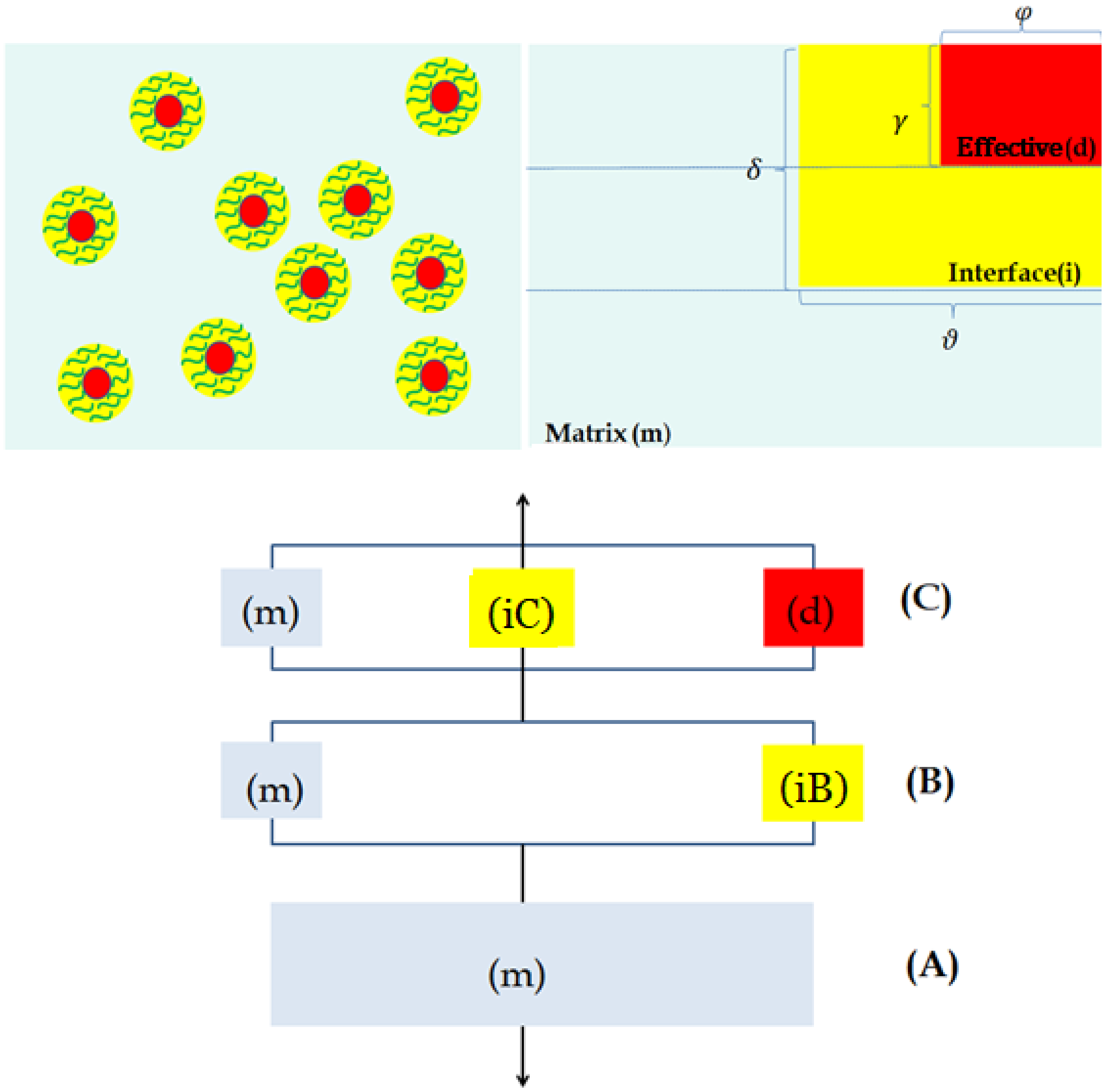

Figure S4 | Three-phase model diagram. Schematic representation of the three-phase model (top) and the series-parallel equivalence diagram (bottom) of the presented approach. The particles are visualized as the red spheres, the particle interphase as the yellow shell having inside polymer chains represented by green lines. The effective numbers of particles contributing to mechanical reinforcement is denoted by the red rectangle.

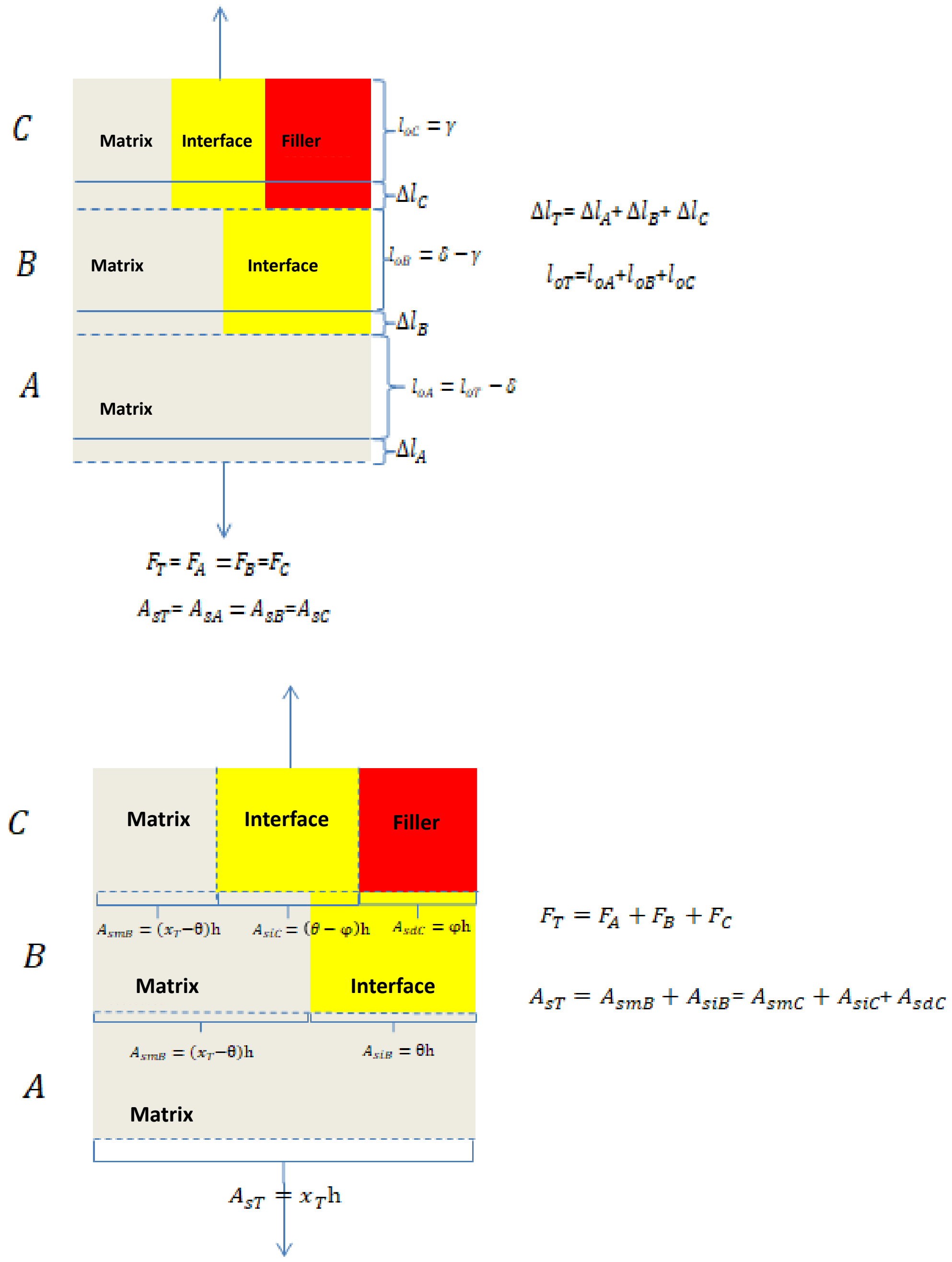

Figure S5 | Three-phase model diagram. Schematic representation of the series-parallel arrangements.

For the case of series connected regions (A, B, C) the following relationships will be fulfilled (see Fig5A):

$$\begin{cases} \Delta l_T = \Delta l_A + \Delta l_B + \Delta l_C \\ F_T = F_A = F_B = F_C \\ A_{sT} = A_{sA} = A_{sB} = A_{sC} \end{cases} \tag{s5.3}$$

Substituting eqs5.2 in eqs5.3 and basis of the geometrical description in FigS.4 yields:

$$\frac{1}{E_T} = \frac{1}{l_{0T}}\left(\frac{l_{0A}}{E_A} + \frac{l_{0B}}{E_B} + \frac{l_{0C}}{E_C}\right) = \frac{1}{l_{0T}}\left(\frac{(l_{0T}-\delta)}{E_m} + \frac{(\delta-\gamma)}{E_B} + \frac{\gamma}{E_C}\right) = \frac{(1-\delta)}{E_m} + \frac{(\delta-\gamma)}{E_B} + \frac{\gamma}{E_C} \tag{s5.4}$$

Because of parallel connected sections, the following relationships can also be obtained (see Fig5B):

$$\begin{cases} F_{TB} = \frac{E_B \Delta l_B A_{sT}}{l_{0B}} = \frac{E_m \Delta l_B A_{smB}}{l_{0B}} + \frac{E_{iB} \Delta l_B A_{siB}}{l_{0B}} \\ F_{TC} = \frac{E_C \Delta l_C A_{sT}}{l_{0C}} = \frac{E_m \Delta l_C A_{smC}}{l_{0C}} + \frac{E_{iB} \Delta l_C A_{siC}}{l_{0C}} + \frac{E_d \Delta l_C A_{sdC}}{l_{0C}} \end{cases} \tag{s5.5}$$

which results in:

$$\begin{cases} E_B = \frac{1}{A_{sT}}(E_m A_{smB} + E_{iB} A_{siB}) \\ E_C = \frac{1}{A_{sT}}(E_m A_{smC} + E_{iC} A_{siC} + E_d A_{sdC}) \end{cases} \tag{s5.6}$$

After expressing the cross sectional area in terms of the length regions (see Fig5B):

$$\begin{cases} E_B = (E_m(1-\theta) + E_{iB}\theta) \\ E_C = (E_m(1-\theta) + E_{iC}(\theta-\varphi) + E_d\varphi) \end{cases} \tag{s5.7}$$

Substituting eqs5.7 in eqs5.4, we obtain:

$$E_T = E_m\left[(1-\delta) + \frac{(\delta-\gamma)}{\left((1-\theta)+\frac{E_{iB}}{E_m}\theta\right)} + \frac{\gamma}{\left((1-\theta)+\frac{E_{iC}}{E_m}(\theta-\varphi)+\frac{E_d}{E_m}\varphi\right)}\right]^{-1} \quad \text{(s5.8)}$$

were the Young modulus of the interfaces B and C are E_{iB} and E_{iC} respectively.

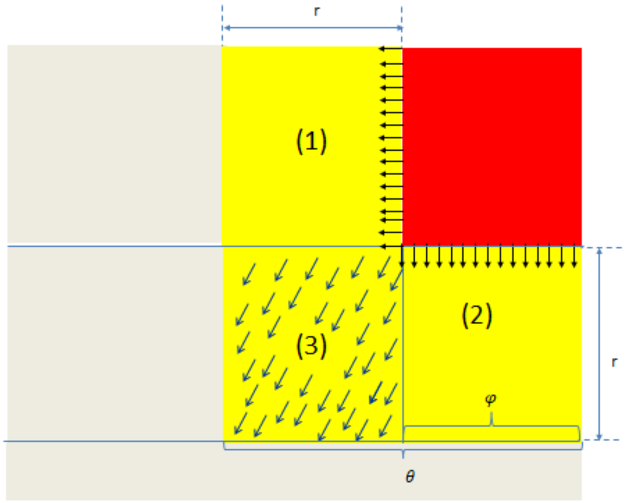

Figure S6 | Three-phase model diagram. Gradient distribution of the different blocks which forms the particle interfaces. Region (3) illustrates the corner effect omitted in X. Ling Ji model.

Calculation of E_{iB} and E_{iC}:

The moduli of the interface in regions B (block2+block3) and C (block 1) are in quite different forms from each other (shown in Fig. S6). The tensile modulus E_{iC} will be that of block 1 (E_{B1}) but the tensile modulus E_{iB} will be calculated as the result of the parallel arrangement between blocks 2 (E_{B3}) and block3 (E_{B3}) which gives rises:

$$E_{iB} = \frac{1}{\theta}\left(E_{B2}\varphi + E_{B3}(\theta-\varphi)\right) \quad \text{(s5.9)}$$

For the calculation of the tensile modulus of each block the following considerations will be taking into account:

- The interface region C and region corresponding to block2 will be analysed as parallel and series arrangement of infinite numbers of volume units.
- In the block1 and block 2, a linear gradient distribution of the modulus along the normal direction of the surface will be assumed and will take the form of the function with a linear gradient decreasing along the normal direction of the surface of dispersed phase.
- In the block 3, a linear gradient distribution of the modulus along the direction of the normalize vector $\vec{u} = \left(-\frac{\sqrt{2}}{2}, -\frac{\sqrt{2}}{2}\right)$ will be assumed and will take the form of the function with a linear gradient decrease along $\vec{u}$.
- The modulus of the interface at the surface of dispersed rigid phase will be assumed as $E_{iC}(0) = E_{iB}(0) = k\, E_m$ where k>0 being E_m the tensile modules

Based on the above mentioned consideration:

$$E_{iC} = \frac{1}{r}\int_0^r E_{iC}(r)dr = -\frac{1}{E_{iC}(0) - E_m}\int_0^r E_{iC}(r)dE_{iC} \quad \text{(s5.10)}$$

After integration,

$$E_{iC} = \frac{1}{E_{iC}(0) - E_m}\frac{E_{iC}(0)^2 - {E_m}^2}{2} = E_m\left(\frac{k+1}{2}\right) \quad \text{(s5.11)}$$

The tensile modules of block2 can then be calculated as:

$$\frac{1}{E_{B2}} = \frac{1}{r}\int_0^r \frac{dr}{E_{iB}(r)} = \frac{1}{E_{iB}(0) - E_m}\int_0^r \frac{dE_{iB}}{E_{iB}(r)} \tag{s5.12}$$

After integration

$$\frac{1}{E_{B2}} = \frac{1}{r}\int_0^r \frac{dr}{E_{iB}(r)} = \frac{1}{E_{iB}(0) - E_m}\left(ln\left(E_{iB}(0)\right) - ln(\,E_m)\right) \tag{s5.13}$$

which yields:

$$E_{B2} = -(E_{iB}(0) - \; E_m)ln\left(\frac{E_m}{E_{iB}(0)}\right) = \; E_m\left(\frac{k-1}{ln[k]}\right) \tag{s5.14}$$

The tensile modules of block3 can be calculated by linear gradient distribution along the normalize vector $\vec{u} = \left(-\frac{\sqrt{2}}{2}, -\frac{\sqrt{2}}{2}\right)$ where x and y axes are defined at the corner between the interception of block1 and block 2 giving rises:

$$E_{B3}(x,y) = E_{B3}(0,0) - \frac{\sqrt{2}}{2}\frac{dE_{B3}}{dx}(x - x_0)\frac{\sqrt{2}}{2}\frac{dE_{B3}}{dy}(y - x_0) \tag{s5.15}$$

After substitution of the directional derivatives:

$$E_{B3}(x,y) = kE_m - \frac{\sqrt{2}}{2}E_m(1-k)\left(\frac{x}{r} + \frac{y}{r}\right) \tag{s5.16}$$

At the point $\left(\frac{1}{2}, \frac{1}{2}\right)$:

$$E_{B3} = E_{B3}\left(\frac{1}{2}, \frac{1}{2}\right) = E_m\left(k + \frac{\sqrt{2}}{2}(k-1)\right) \tag{s5.17}$$

Substituting eqs5.17 and eqs5.14 in eqs5.9

$$E_{iB} = \frac{E_m}{\delta}\left(\left(\frac{k-1}{ln[k]}\right)\varphi + \left(\left(k + \frac{\sqrt{2}}{2}(k-1)\right)(\theta - \varphi)\right)\right) \quad \text{(s5.18)}$$

Substituting eqs5.18 and eqs5.11 in eqs5.8 and considering particles random orientations we get the eq.8a of the paper:

$$E_c = E_m \left[(1-\delta) + \frac{\delta-\gamma}{(1-\delta)+\left(\frac{k-1}{ln[k]}\right)\gamma+\left(\left(k+\frac{\sqrt{2}}{2}(k-1)\right)(\delta-\gamma)\right)} + \frac{\gamma}{(1-\delta)+\frac{(\delta-\gamma)(k+1)}{2}+\gamma\frac{E_f}{E_m}}\right]^{-1} \quad \text{(s5.19)}$$

Here it is important to remark that eq s5.19 has a corrective term, which corrects eq.4 of the paper.

Because the dispersed phase is in spherical form and hence random orientations can be considered deriving the following relationship:

$$\begin{cases} \gamma^2 = \phi_{eff} \\ \delta = \sqrt{\left(1+\frac{r}{R}\right)\phi_{eff}} \end{cases} \quad \text{(s5.20)}$$

Substituting eqs5.20 in eq.5.19 and considering the definition of effective fraction of particles (eqs2.3), we get the eq.8 of the paper:

$$E_c = E_m \left[(1-\delta) + \frac{\delta-\gamma}{(1-\delta)+\left(\frac{k-1}{ln[k]}\right)\gamma+\left(\left(k+\frac{\sqrt{2}}{2}(k-1)\right)(\delta-\gamma)\right)} + \frac{\gamma}{(1-\delta)+\frac{(\delta-\gamma)(k+1)}{2}+\gamma\frac{E_f}{E_m}}\right]^{-1} \quad \text{(s5.21)}$$

$$\delta = \begin{cases} 0 & : 0 \le \phi \le \phi_p \\ \sqrt{\left(1+\frac{r}{R}\right)\phi_g}\left(\frac{\phi-\phi_p}{\phi_g-\phi_p}\right)^{\alpha/2} & : \phi_p < \phi \le \phi_g \end{cases} \quad \text{(s5.22)}$$

$$\gamma = \begin{cases} 0 & : 0 \leq \phi \leq \phi_p \\ \left[\sqrt{\phi_g}\left(\frac{\phi-\phi_p}{\phi_g-\phi_p}\right)\right]^{\alpha/2} & : \phi_p < \phi \leq \phi_g \end{cases} \quad \text{(s5.23)}$$

References

1. Ning Ren et al, Filler-Reinforced Elastomers Based on Functional Polyolefin Prepolymers Ind. Eng. Chem. Res. 2016, 55, 6106−6112
2. M. Kuo, C. Tsai, J. Huang and M. Chen, PEEK composites reinforced by nano sized SiO2 and Al2O3 particulates, Mater. Chem. Phys., 2005, 90, 185–195
3. M. I. Sarwar, S. Zul qar and Z. Ahmad, Preparation and properties of polyamide–titania nanocomposites, J. Sol-Gel Sci. Technol., 2007, 44, 41–46.
4. X. Wang, L. Wang, Q. Su and J. Zheng, use of unmodified SiO2 as nanofiller to improve mechanical properties of polymer-based nanocomposites, Compos. Sci. Technol., 2013, 89, 52–60
5. T. Schilling, M.A. Miller, P. van der Schoot, Europhys. Lett. 2015, 111, 56004.
6. X. L. Ji, J. K. Jing, W. Jiang and B. Z. Jiang, Tensile modulus of polymer nanocomposites, Polym. Eng. Sci., 2002, 42, 983– 993M.
